# Analysis of the lncRNA-Associated Competing Endogenous RNA (ceRNA) Network for Tendinopathy

**DOI:** 10.1155/2022/9792913

**Published:** 2022-05-12

**Authors:** Bing-Zhe Huang, Yang Jing-Jing, Xiao-Ming Dong, Zhuan Zhong, Xiao-Ning Liu

**Affiliations:** ^1^Orthopaedic Medical Center, The Second Hospital of Jilin University, No. 218, Ziqiang Street, Changchun, 130041, Jilin, China; ^2^Operation Service, The Second Hospital of Jilin University, No. 218, Ziqiang Street, Changchun, 130041, Jilin, China

## Abstract

**Background:**

We aimed to construct the lncRNA-associated competing endogenous RNA (ceRNA) network and distinguish feature lncRNAs associated with tendinopathy.

**Methods:**

We downloaded the gene profile of GSE26051 from the Gene Expression Omnibus (GEO), including 23 normal samples and 23 diseased tendons. Differentially expressed mRNAs (DEmRNAs) and differentially expressed lncRNAs (DElncRNAs) were identified, and functional and pathway enrichment analyses were performed. Protein-protein interaction (PPI) network was constructed and further analyzed by module mining. Moreover, a ceRNA regulatory network was constructed based on the identified lncRNA–mRNA coexpression relationship pairs and miRNA–mRNA regulation pairs.

**Results:**

We identified 1117 DEmRNAs and 57 DElncRNAs from the GEO data. The downregulated DEmRNAs were particularly associated with muscle contraction and muscle filament, while the upregulated ones were linked to extracellular matrix organization and cell adhesion. From the PPI network, 11 modules were extracted. Genes in MCODE 2 (such as TPM4) were significantly involved in cardiomyopathy, and genes in MCODE 4 (such as COL4A3 and COL4A4) were involved in focal adhesion, ECM-receptor interaction, and PI3K-Akt signaling pathway. The ceRNA network contained 7 lncRNAs (MIR133A1HG, LINC01405, PRKCQ-AS1, C10orf71-AS1, MBNL1-AS1, HOTAIRM1, and DNM3OS), 63 mRNAs, and 41 miRNAs. Downregulated lncRNA MIR133A1HG could competitively bind with hsa-miR-659-3p and hsa-miR-218-1-3p to regulate the TPM3. Meanwhile, MIR133A1HG could competitively bind with hsa-miR-1179 to regulate the COL4A3. Downregulated C10orf71-AS1 could competitively bind with hsa-miR-130a-5p to regulate the COL4A4.

**Conclusions:**

Seven important lncRNAs, particularly MIR133A1HG and C10orf71-AS1, were found associated with tendinopathy according to the lncRNA-associated ceRNA network.

## 1. Introduction

Tendinopathy is characterized by a disorganized, haphazard healing response with no histological signs of inflammation. [1] Pathologies of the Achilles, patellar, and rotator cuff tendons are still common problems for recreational and competitive athletes and for individuals lacking any history of promoted physical activity. [2] A particular problem is rotator cuff tendinopathy (RCT), which is a common musculoskeletal disorder, accounting for 85% of shoulder joint pain. [3] Extrinsic factors result in a large number of acute tendon injuries, such as tendinopathy. Although in overuse syndromes, the combination of intrinsic factors may be the cause of tendinopathy, such as age-related changes in cell activity and external factors.

Recently, research focus has been placed on long noncoding RNAs (lncRNAs), which are a category of RNA molecules of over 200 nucleotides in length with an inconspicuous open reading frame. [4] Previous studies have reported that many human diseases, such as orthopedic and cancer diseases, were associated with deficiency, mutation, or overexpression of lncRNAs. Salmena et al. [5] proposed the competing endogenous RNA (ceRNA) hypothesis in 2011. Subsequently, it was asserted that ceRNAs play roles in orthopedic diseases, such as RCT and osteoarthropathy (OA). Ge et al. [3] identified several lncRNAs and mRNAs, such as COL11A1 and COL2A1, which show differential expression in RCT, suggesting their relation to the process of this disorder. In addition, Shen et al. [6] reported that SNHG5 refers to the mechanism of OA via acting as a ceRNA to competitively sponge miR-26a, thereby regulating the expression of SOX2. Moreover, Li et al. [7] demonstrated that the lncRNA XIST could increase OA chondrocytes proliferation and increase apoptosis via the miR-211/CXCR4 axis. Therefore, the lncRNA XIST is a valuable curative target for treating OA. These researches express that other lncRNAs might also occupy vital roles in the etiopathogenesis of orthopedic diseases.

In the study of Jelinsky et al. [8], the differentially expressed mRNAs (DEmRNAs) between normal and tendinopathy samples (mainly RCT tear and extensor carpi radialis brevis tear) were revealed, and pathway analysis was conducted, and the results showed that tendinopathy samples lacked appreciable inflammatory response and had altered expressions of extracellular matrix proteins. Thereafter, Cai et al. downloaded the GSE26051 that was deposited by Jelinsky et al., the potential regulatory microRNA of DEmRNAs was further predicted, and especially, miR-499 was found to regulate specific genes, including CUGBP2 and MYB [9]. Previous studies contribute to the understanding of molecular mechanisms of tendinopathy. However, to the best of our knowledge, the roles of ceRNAs in the pathologies of tendinopathy have seldomly been investigated. In order to further elucidate the roles of lncRNAs in tendinopathy, we downloaded GSE26051 that deposited by Jelinsky et al. from Gene Expression Omnibus (GEO). The differentially expressed mRNAs (DEmRNAs) and differentially expressed lncRNAs (DElncRNAs) in tendinopathy samples were identified, followed by functional and pathway enrichment analyses. Protein-protein interaction (PPI) was constructed for module analysis. Moreover, an lncRNA-associated ceRNA regulatory network was constructed based on the lncRNA–mRNA coexpression relationship pairs and miRNA–mRNA regulation pairs. Hopefully, the results of our study could assist in understanding the roles of lncRNAs in tendinopathy and provide valuable treatment targets.

## 2. Material and Methods

### 2.1. Affymetrix Microarray Dataset

Gene expression profilings of tendinopathy were retrieved from National Center for Biotechnology Information (NCBI) GEO (https://www.ncbi.nlm.nih.gov/geo/) by searching the keywords of “*Homo sapiens*” and “chronic tendon injuries”. The inclusion criteria of datasets were as follows: samples were collected from patients with chronic tendon injuries and contained normal samples; the number of overall samples was not less than 20. One dataset GSE26051 [8] meeting the above criteria was downloaded from GEO [10] (http://www.ncbi.nlm.nih.gov/geo/), which was based on the GPL570 [HG-U133_Plus_2] Affymetrix Human Genome U133 Plus 2.0 Array platform. This dataset consisted of 46 specimens including 23 biopsies of diseased tendons and 23 biopsies of normal-appearing tendons from 23 patients who were undergoing surgical procedures for the treatment of chronic tendinopathy.

### 2.2. Data Preprocessing and Annotation

We analyzed the derived genetic data using the affy package in R [11] (version 1.50.0, http://www.bioconductor.org/packages/release/bioc/html/affy.html). Then, we used the RMA (robust multiarray average) method to perform background correction, data normalization, and expression calculation. We obtained the expression values of probes in the normal and diseased tendons.

Subsequently, we obtained the sequence corresponding to the HG-U133_Plus_2 probe from the platform annotation file. We downloaded the current human reference genome (GRCh38) from the GENCODE database [12] (https://www.gencodegenes.org/releases/current.html). Then, we used SeqMap [13] to map all of the probe sequences to the reference genome. The unique mapping probes were retained, and then, the gene corresponding to each probe was obtained on the basis of the human gene annotation file (Release 25) provided by GENCODE according to the genomic features such as position in chromosome, sense, or antisense direction. Protein-coding probes were considered as probes of mRNAs, and probes with the annotation features of antisense, sense_intronic, lincRNA, sense_overlapping, or processed_transcript were considered as probes of lncRNAs.

### 2.3. Differential Expression Analysis

The Bayes method in the limma package (version 3.34.7, https://bioconductor.org/packages/release/bioc/html/limma.html) was used to conduct the differential expression analysis. The DEmRNAs and DElncRNAs between the normal and diseased tendons were uncovered with the thresholds of *p* value < 0.05 and |log2 fold change (FC)| > 0.585. The DElncRNAs were recorded as featured lncRNAs (lncRNAs with functional annotation and literature support) in the LncBook [14] database (http://bigd.big.ac.cn/lncbook/index) or LNCiperia [15] database (https://lncipedia.org/) that were retained for subsequent analysis.

### 2.4. GO BPs and Pathway Enrichment Analyses for DEmRNAs

Analysis of the GO BP [16] and KEGG pathway enrichment annotations was performed for DEmRNAs on basis of The Database for Annotation, Visualization, and Integrated Discovery [12] (DAVID, version 6.8, https://david.ncifcrf.gov/) [17,18], with *p* < 0.05 and count ≥2 as the thresholds.

Moreover, GO and KEGG pathway enrichment analyses were carried out using the online tool Metascape (http://metascape.org). [19] Parameters were set to min overlap = 3; *p* value = 0.01; min enrichment = 1.5; and enrichment factor >1.5. The enrichment factor is the ratio between the observed count and the accidentally expected count. After obtaining the terms that fulfill the above criteria, clustering analysis was carried out according to the similarity of genes (similarity >0.3) enriched in each term. The most statistically significant term with the minimum *p* value was considered as a representative of that cluster. The top 20 most significant clusters were retained for bar graph display.

### 2.5. PPI Network and Analysis of Module Mining

The online tool Metascape was applied to mine the PPI relationships of the above-mentioned differentially expressed genes. Then, the databases BioGRID [20], inweb_ IM [21], and OmniPath [22] were used for protein-protein interaction enrichment analysis, using the default parameters (min network size = 3; max network size = 500). After getting the PPI pairs, the network was visualized using the software of Cytoscape (version 2.1.6, http://apps.cytoscape.org/), and the plugin CytoNCA (http://apps.cytoscape.org/apps/cytonca) [23] was used to analyze the degree of network nodes. The parameter was set without weighting. Through ranking the connectivity of each node, we obtained the important nodes in the PPI network, namely the hub proteins. Furthermore, we used the Metascape tool to mine PPI network modules based on the molecular complex detection (MCODE) algorithm. [24] At the same time, we performed GO BP (GO biological process) and KEGG pathway enrichment analyses for each module.

### 2.6. Analysis of Coexpression of lncRNA and mRNA and Pathway Enrichment Analysis of lncRNA

The Pearson correlation coefficients of the above-mentioned DEmRNAs and DElncRNAs were calculated, and the correlation test was carried out using the matched mRNA and lncRNA data. In addition, Benjamini-Hochberg (BH) procedure was used to obtain the adjusted *p* value [25]. To construct the subsequent ceRNA network, we focused on the pairs with correlation coefficient r >0.7 and adjusted *p* value < 0.05.

The KEGG pathway enrichment analysis was performed for the target genes of each lncRNA that was enriched by using clusterProfiler in R (version: 3.8.1, http://bioconductor.org/packages/release/bioc/html/clusterProfiler.html) [26], and the adjusted *p* value was obtained by BH procedure. Significantly enriched pathways were obtained with the threshold of the adjusted *p* value < 0.05. The top ten lncRNA pathways were displayed in a bubble chart.

### 2.7. The miRNA Prediction Analysis

On basis of the mRNAs in the constructed lncRNAmRNA coexpression network, the miRWalk 2.0 [27] (http://zmf.umm.uni-heidelberg.de/apps/zmf/mirwalk2/) database was used to obtain miRNA–mRNA pairs that simultaneously recorded in miRWalk, Miranda, miRDB, RNA22, and TargetScan. Then, on basis of the lncRNAs in the lncRNA–mRNA coexpression network, the online database lncbasev2 [28] (http://carolina.imis.athena-innovation.gr/diana_tools/web/index.php?r=lncbasev2%2Findex) was used to predict the lncRNA–miRNA relationship pairs with the cutoff criterion of score >0.95.

### 2.8. Construction of the ceRNA Network

Based on the miRNA–mRNA and lncRNA–miRNA relationship pairs obtained as described above, we first selected the lncRNA–miRNA–mRNA relationship pairs regulated by the same miRNA and then performed further lncRNA–miRNA–mRNA screening by combining the positive coexpression relationship between mRNA and lncRNA (correlation coefficient *r* > 0.7). The lncRNAs and mRNAs regulated by the same miRNAs in the ceRNA network that have a positive coexpression relationship were considered as ceRNAs. Finally, we used the Cytoscape plugin CycloNCA to analyze the node degree, and the parameter was set without weighting. A higher connection degree reflects the greater importance of the node in the network.

## 3. Results

### 3.1. Identification of DEmRNAs and DElncRNAs in Tendinopathy

**Figure 1:**
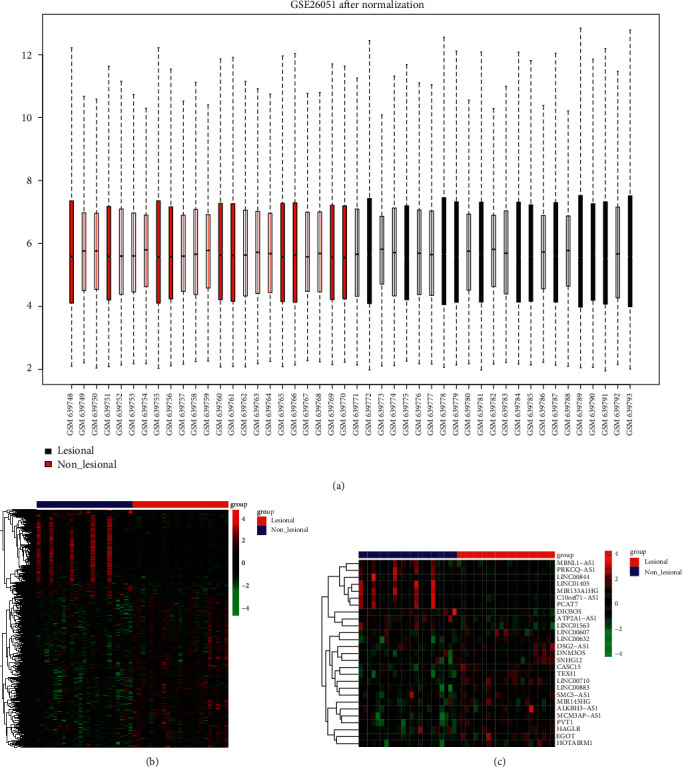
Identification of DEmRNAs and DElncRNAs. (a) Box graph of expression level distribution of sample genes. (b) Heatmap of DEmRNAs. (c) Heatmap of DElncRNAs.

A total of 37,666 probes were obtained through data preprocessing and standardization, which is shown in Appendix Table 1. The box diagram of the gene expression distribution of each sample is shown in Figure 1(a), and the median value of sample gene expression was basically at the same level, which could be directly used for subsequent difference analysis. When compared with normal controls, a total of 1507 probes were regarded as differentially expressed in tendinopathy samples, which corresponded to 1117 DEmRNAs (including 702 upregulated DEmRNAs and 415 downregulated ones) and 57 DElncRNAs (including 29 upregulated DElncRNAs and 28 downregulated ones) (Table 1). Twenty-seven lncRNAs were screened from the featured lncRNAs in the LncBook database or lncRNAs recorded in the LNCiperia database (Appendix Table 2). Based on the expressions of the DEmRNAs and 27 functional lncRNAs, the heatmaps of DEmRNAs (Figure 1(b)) and functional lncRNAs (Figure 1(c)) showed that the samples were clustered in two different directions.

### 3.2. Gene Function and Pathway Enrichment Analyses

**Figure 2:**
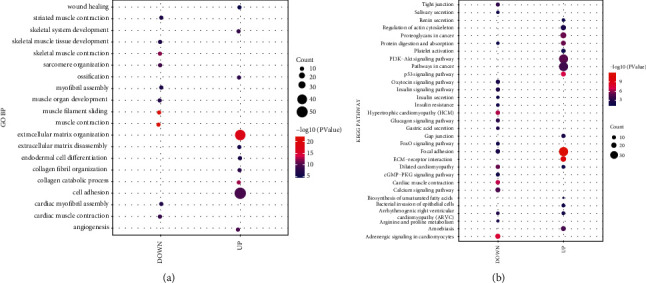
GO BP and KEGG pathway enrichment analyses for DEmRNAs. The top 10 GO BP (a) and KEGG pathway. (b) Enrichment analysis results of upregulated or downregulated DEmRNAs.

Totally, 148 GO BPs and 16 KEGG pathways were highly enriched with a cutoff of *p* value < 0.05 for the upregulated DEmRNAs, while 79 GO BPs and 19 KEGG pathways were obtained for the downregulated DEmRNAs. The top 10 GO BPs and KEGG pathways (ranked by *p* value) are shown in Figure 2.  Figure 2(a) reveals that the downregulated DEmRNAs were particularly associated with muscle contraction and muscle filament, while the upregulated DEmRNAs were particularly associated with extracellular matrix organization and cell adhesion. Furthermore, the KEGG pathway enrichment analysis findings indicated that the most remarkable pathway was focal adhesion (Figure 2(b)).

**Figure 3:**
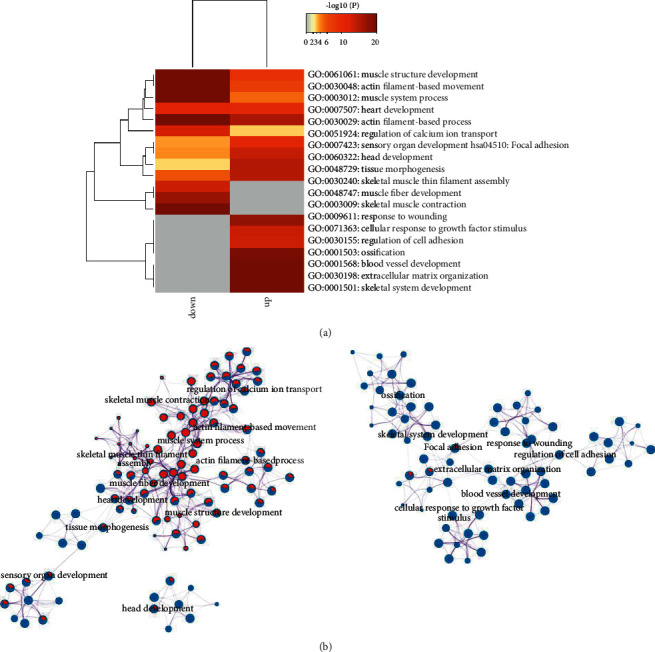
GO BP and KEGG pathways of upregulated and downregulated mRNAs. (a) Bar graph of the top 20 clusters. (b) The relationships among the enriched clusters from the KEGG analysis were visualized using Metascape.

Besides, through the KEGG pathways enrichment analysis and GO BPs in the Metascape database, five pathways involved in the downregulated DEmRNAs included muscle structure development, actin filament-based movement, muscle system process, actin filament-based process, and skeletal muscle contraction. Besides, four pathways shown to be related to the upregulated DEmRNAs were ossification, blood vessel development, extracellular matrix organization, and skeletal system development (Figure 3(a)).

Moreover, term-term interaction network (Figures 3(b)) showed that the upregulated and downregulated DEmRNAs participate in different functions. The network consists of 172 nodes, representing 172 terms, and 947 edges were enriched, which represent the similarity score between terms. Metascape obtained the similarity score between the two terms. The larger the score, the thicker the connection lines.

### 3.3. Construction of PPI Network and Module Mining Analysis

**Figure 4:**
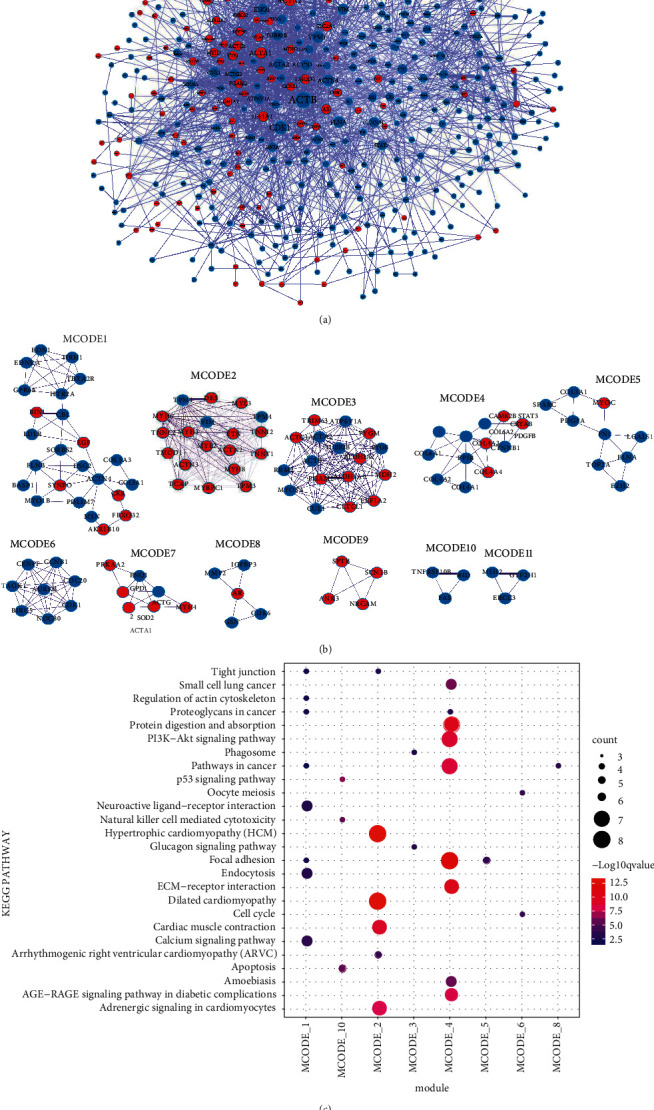
Construction of PPI network and module mining analysis. (a) PPI network analysis. (b) PPI network submodule. (c) Enrichment analysis results of the submodule KEGG pathway in the PPI network.

A total of 2095 PPI pairs, including 440 DEmRNAs, were identified to construct the PPI network (Figure 4(a)). In the PPI network, the top 10 genes ranked by node degree were ACTB, CDK1, ACTN2, ACTA1, TPM1, ACTA2, ACTN4, FLNA, ENO1, and ACTN1 in order. Moreover, 11 module substructures were distinguished in the PPI network (Figures 4(b)). The pathway enrichment analysis for the genes in the 11 modules showed that genes in MCODE 2 (DES, MYH6, MYL2, MYL3, TPM1, TPM3, TPM4, and TTN) were significantly involved in dilated cardiomyopathy and hypertrophic cardiomyopathy (HCM), and genes in MCODE 4 (COL4A1, COL4A2, COL4A3, COL4A4, COL6A1, COL6A2, CTNNB1, and PDGFB) were mainly involved in focal adhesion, ECM-receptor interaction, and PI3K-Akt signaling pathway (Figure 4(c), Appendix Table 3).

### 3.4. Coexpression Analysis of DEmRNAs and DElncRNAs

**Figure 5:**
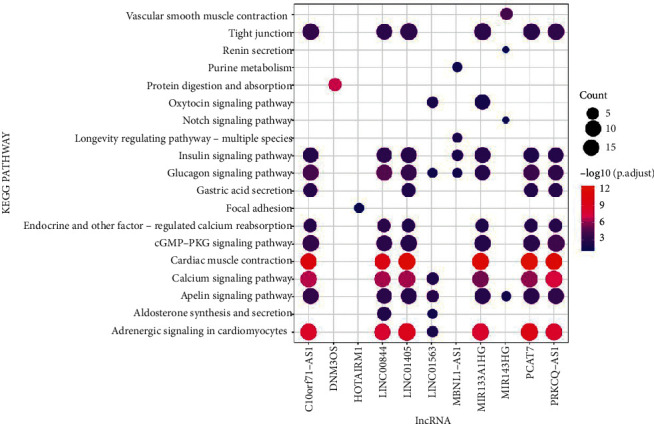
KEGG pathway analysis of lncRNA target genes.

A total of 1851 significant coexpression relationships were screened with a cutoff of *p* < 0.05, which included 422 mRNAs and 22 lncRNAs. KEGG pathways were enriched for the target genes of 11 lncRNAs among the 22 lncRNAs, and the top ten KEGG pathways are shown in Figure 5. The results showed that the target genes of C10orf71−AS1, LINC00844, LINC01405, MIR133A1HG, PCAT7, and PRKCQ−AS1 were particularly associated with cardiac muscle contraction, followed by adrenergic signaling in cardiomyocytes and calcium signaling pathway (Figure 5).

### 3.5. Examining the Potential Regulatory miRNAs and Construction of the ceRNA Network

**Figure 6:**
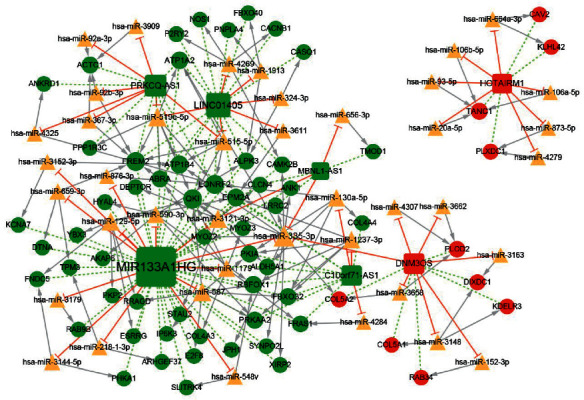
The differentially expressed subnetwork of the ceRNA network in tendinopathy. The red and green circles represent upregulated and downregulated mRNAs, respectively.

In total, 201 lncRNA–miRNA pairs were predicted for the 11 lncRNAs whose target genes were enriched in KEGG pathways, including 184 miRNAs and 10 lncRNAs. In addition, based on the 422 mRNAs in the lncRNA–mRNA coexpression network, a total of 3389 miRNA–mRNA pairs were predicted using miRWalk, including 961 miRNAs and 233 mRNAs. Then, based on the above-mentioned lncRNA–miRNA and miRNA–mRNA relationship pairs, we screened the miRNA–lncRNA–mRNA relationship pairs regulated by the same miRNA and combined the positive coexpression relationship between mRNA and lncRNA (correlation coefficient *r* > 0.7). Then, we further screened the lncRNA–miRNA–mRNA relationship pairs and established the network, namely, the ceRNA network (Figure 6). The ceRNA network comprised 46 lncRNA–miRNA pairs, 114 miRNA–mRNA pairs, and 87 lncRNA–mRNA pairs, involving 7 lncRNAs, 63 mRNAs, and 41 miRNAs (Appendix Table 4). Among the 7 lncRNAs, there were 5 downregulated lncRNAs (MIR133A1HG, LINC01405, PRKCQ-AS1, C10orf71-AS1, and MBNL1-AS1) and 2 upregulated ones (HOTAIRM1 and DNM3OS). These lncRNAs ranked by descending node degree were MIR133A1HG, LINC01405, PRKCQ-AS1, C10orf71-AS1, DNM3OS, HOTAIRM1, and MBNL1-AS1 in order. Downregulated MIR133A1HG could competitively bind with 13 miRNAs, including hsa-miR-659-3p and hsa-miR-218-1-3p to regulate the TPM3. Meanwhile, MIR133A1HG could competitively bind with hsa-miR-1179 to regulate the COL4A3. Downregulated C10orf71-AS1 could competitively bind with hsa-miR-130a-5p to regulate the COL4A4.

## 4. Discussion

In our study, our purpose was to distinguish potential lncRNAs and mRNAs involved in tendinopathy. Overall, data from GEO revealed 1117 DEmRNAs and 57 DElncRNAs. Next, we identified the function and signaling pathways, where these DEmRNAs and lncRNAs were embroiled. What's more, we established a ceRNA coexpression network that contained 7 lncRNAs, 63 mRNAs, and 41 miRNAs using bioinformatic tools.

Recently, increasing evidence has indicated that lncRNA participates in tendinopathy. Zhang et al. [29] detected 40 different expressed lncRNAs in tendinopathy, such as LOC100507027. Then, Ge et al. [3] constructed a coexpressed network comprising the vital genes including NONHSAT209114.1, ENST00000577806, NONHSAT168464.1, PLK2, TMEM214, and IGF2 for tendinopathy. Furthermore, a mechanism study demonstrated that H19 promotes tenogenic differentiation both in vitro and in vivo by targeting miR-29b-3p and activating TGF-*β*1 signaling [30]. As previous study indicated that lncRNAs also played a key role in male infertility [31], Rotondo et al. [32] reported that lncRNA H19 was dysregulated in male infertility by epigenetic impairments. The defective marks of H19 could be potentially employed as useful tools in clinical practice for assessing male infertility [33]. Nafiseh et al [34] found that SLC7A11-AS1 played a potential role in the pathophysiology of male infertility associated with varicocele. These reports indicated that lncRNA expression could regulate the development of some diseases. Further experimental and functional studies need to be performed for exploring the role of lncRNAs. When such studies are done, these findings should help guide them in the future. The important BPs and pathways in tendinopathy were identified using GO and KEGG pathway analyses on basis of 1117 DEmRNAs. Most of the BPs and pathways play a crucial role in tendinopathy, for example, muscle contraction, muscle filament, matrix organization, cell adhesion, and focal adhesion. [35] Besides, eleven intersecting lncRNAs that overlap between the GO and KEGG pathway analyses were identified in tendinopathy. The results showed that C10orf71−AS1, LINC00844, LINC01405, MIR133A1HG, PCAT7, and PRKCQ−AS1 were particularly associated with cardiac muscle contraction, followed by adrenergic signaling in cardiomyocytes. Previous studies indicated that cardiac muscle contraction is involved in diabetes and ischemia/reperfusion oxidative injury. [36]

Moreover, a total of 2095 PPI pairs, including 440 DEmRNAs, were contained in the PPI network. In total, 10 hub genes, namely, ACTB, CDK1, ACTN2, ACTA1, TPM1, ACTA2, ACTN4, FLNA, ENO1, and ACTN1, were screened from the PPI network. Tsai et al. [37] indicated that geranylgeranyl pyrophosphate could prevent the adverse effect of simvastatin in tendon cells through downregulating CDK1 expression. In contrast, these other hub genes have not been reported to be related to tendinopathy. Moreover, 11 modules were extracted from the PPI network. The results showed that genes (DES, MYH6, MYL2, MYL3, TPM1, TPM3, TPM4, and TTN) in MCODE 2 were significantly involved in dilated cardiomyopathy and hypertrophic cardiomyopathy (HCM). It has been reported that rupture of the distal biceps tendon was found in 33.3% of patients with wild-type transthyretin amyloidosis cardiomyopathy [38]. Meanwhile, genes (COL4A1, COL4A2, COL4A3, COL4A4, COL6A1, COL6A2, CTNNB1, and PDGFB) in MCODE 4 were involved in focal adhesion, ECM-receptor interaction, and PI3K-Akt signaling pathway. The transcription factor scleraxis (Scx) plays a critical role in tenocyte mechanotransduction, and focal adhesions and extracellular matrix-receptor interaction were significantly downregulated in Scx knockdown tenocytes [39]. Inhibition of adipogenesis of tendon stem cells and fatty infiltration in injury tendon could promote biomechanical properties and decrease rupture risk of injury tendon by downregulating PTEN/PI3K/AKT signaling [40]. Thus, genes in MODE 2 and MODE 4 might be associated with tendinopathy by regulating cardiomyopathy, focal adhesion, ECM-receptor interaction, and PI3K-Akt signaling pathway.

Moreover, on basis of the lncRNA–mRNA coexpression relationship pairs and miRNA–mRNA regulation pairs, a lncRNA-associated ceRNA network was constructed. In the ceRNA network, there were 7 lncRNAs, 63 mRNAs, and 41 miRNAs. Downregulated lncRNA MIR133A1HG could competitively bind with hsa-miR-659-3p and hsa-miR-218-1-3p to regulate the expression of TPM3 and might influence the cardiomyopathy. Meanwhile, MIR133A1HG could competitively bind with hsa-miR-1179 to regulate the expression of COL4A3, and downregulated C10orf71-AS1 could competitively bind with hsa-miR-130a-5p to regulate the expression of COL4A4, thereby might regulate the focal adhesion, ECM-receptor interaction, and PI3K-Akt signaling pathway.

However, our study still has some limitations. Firstly, the main limitation of the study is the lack of validation in vitro/vivo as well as functional validation on the role of lncRNAs and mRNAs in tendinopathy with cell lines. Then, the sample size of our study was small in the computational analyses, and bigger samples were necessary to validate these lncRNAs' functions in tendinopathy.

## 5. Conclusion

In conclusion, a lncRNA-associated ceRNA regulatory network was constructed, and seven important lncRNAs were identified associated with tendinopathy. MIR133A1HG may influence the expressions of TPM3 and COL4A3 by competitively binding with hsa-miR-218-1-3p and hsa-miR-1179. C10orf71-AS1 may influence the expression of COL4A4 by competitively binding with hsa-miR-130a-5p.

## Figures and Tables

**Table 1:** Statistics of number of differentially expressed genes (probes).

	mRNA	lncRNA
Up	702 (959)	29 (29)
Down	415 (548)	28 (30)
Total	1117 (1507)	57 (59)

*Note.* The number outside the bracket is the horizontal number of genes, and the number inside the bracket is the number of corresponding probes.

## Data Availability

The dataset used and/or analyzed during the current study are available from the corresponding author upon reasonable request.

## Abbreviations

DEmRNAs: Differentially expressed mRNAs
DElncRNAs: Differentially expressed long noncoding RNAs
ceRNA: Competing endogenous. RNA
DEmRNAs: Differentially expressed mRNAs
DElncRNAs: Differentially expressed long noncoding RNAs.
GO BPs: Gene Ontology biological processes
KEGG: Kyoto Encyclopedia of Genes and Genomes
PPI: Protein-protein interaction.

## Supplementary Materials

Supplementary Materials Table 1 shows data preprocessing and standardization. Table 2 shows featured lncRNAs in the LncBook database or lncRNAs recorded in the LNCiperia database. Table 3 shows pathway enrichment analysis for the genes in the 11 modules. Supplementary Materials
